# Editorial for the Special Issue: Epidemiology, Transmission, Cell Biology and Pathogenicity of *Cryptosporidium*

**DOI:** 10.3390/microorganisms9030511

**Published:** 2021-03-01

**Authors:** Gabriela Certad, Eric Viscogliosi

**Affiliations:** 1 University Lille, CNRS, Inserm, CHU Lille, Institut Pasteur de Lille, U1019—UMR 9017—CIIL—Centre d’Infection et d’Immunité de Lille, F-59000 Lille, France; eric.viscogliosi@pasteur-lille.fr; 2 Délégation à la Recherche Clinique et à l’Innovation, Groupement des Hôpitaux de l’Institut Catholique de Lille, F-59462 Lomme, France

The apicomplexan parasite Cryptosporidium represents a major public health problem in humans and animals by causing self-limited diarrhea in immunocompetent individuals and life-threatening disease in immunocompromised hosts. In this regard, recent data from the Global Enteric Multicenter Study (GEMS) reported that *Cryptosporidium* is among the leading global causes of moderate to severe diarrhea in children aged less than 2 years. However, despite its significant impact on public health, dynamics of *Cryptosporidium* infection remain to be further investigated and currently, no fully effective treatment or vaccines are available. In addition, several pieces of evidence support an association between *Cryptosporidium* and the development of digestive neoplasia. Interestingly, this parasite is ubiquitous and has been isolated from a wide range of hosts. In addition, oocyst stages of *Cryptosporidium* can survive for months in the environment, being resistant to most chemical disinfectants, and the main cryptosporidiosis outbreaks are caused by contaminated water, even if zoonotic and foodborne transmission have also been commonly reported in the literature. The low dose of oocysts required for infection also promotes the circulation and spread of the parasite.

In spite of some recent significant advances, many questions persist regarding epidemiology, transmission and pathogenicity of *Cryptosporidium*. Therefore, the main objective of this special issue including 14 papers was to expand our knowledge regarding different biological aspects of the parasite.

The first surveys of this special issue focused on the epidemiology, genetic diversity and transmission of *Cryptosporidium*. Wells et al. [1] reported data regarding the identification and prevalence of *Cryptosporidium* species and subtypes in calves and geese co-grazing on livestock farms surrounding reservoirs of water supplies intended for human consumption in Mainland Orkney (Scotland). The high prevalence of *C. parvum* found in calves, geese and water samples represented a significant risk to water quality and public health. The article by Wang et al. [2] was related to avian *Cryptosporidium* isolates, being the first report of the parasite in whooper swans in China. *Cryptosporidium* was detected in 1.7% of the samples tested, with the presence of two zoonotic species (*C. parvum* and *C. andersoni*) and one avian genotype (*Cryptosporidium* goose genotype II). The research article by Li et al. [3] provided information about the prevalence and diversity of species and genotypes of *Cryptosporidium* in the resident and migratory wildlife populations located in a major, protected watershed in the Pacific Northwest. The overall prevalence of *Cryptosporidium* in the wildlife scat reached 13.8%, with 15 species of wildlife found to be positive for the parasite. Molecular analysis showed that several species and genotypes of *Cryptosporidium* were present, with some isolates possibly co-circulating within and between wildlife populations. Moreover, evidence of oocyst exchange between infected prey and their predators was confirmed together with the identification of a range of *Cryptosporidium* isolates with varying levels of zoonotic potential. Another epidemiological survey focusing on wildlife was carried out by Certad et al. [4]. In a previous work from the same authors, six novel genotypes of *Cryptosporidium* colonizing marine fish were identified based on the 18S rDNA locus. Of these six genotypes, five grouped together while the last one emerged separately. In the current paper, phylogenetic analysis of these same genotypes at the actin locus confirmed that these two clusters were representative of potential novel *Cryptosporidium* fish species. In their paper, Yang et al. [5] described the identification of the 60-kDa glycoprotein (*gp60)* gene of *C. ryanae* in a whole-genome sequence for the further development of a subtyping tool. The use of this tool in the characterization of isolates from dairy cattle, beef cattle, yaks, and water buffaloes highlighted a high genetic diversity in *C. ryanae*. The data obtained also provided evidence for the likely occurrence of host adaptation and geographical differences in the distribution of *C. ryanae* subtypes. On the whole, these five first papers demonstrated that livestock (mainly cattle) and wildlife are relevant contributors to the zoonotic transmission of *Cryptosporidium.*

The Special Issue also included two articles concerning the epidemiology of cryptosporidiosis in humans, showing that even in high-income countries, cryptosporidiosis diagnosis and surveillance are required. Muadica et al. [6] conducted a PCR and sequencing-based study investigating the occurrence and molecular diversity of intestinal protozoa including *Cryptosporidium* among a population of asymptomatic schoolchildren in Madrid, Spain, and revealed a low prevalence of *Cryptosporidium* spp. (0.9%) in this cohort, in link with a large anthroponotic transmission. The second survey by Costa et al. [7] presented epidemiological data obtained in France from 2017 to 2019 by the National Reference Center—Expert Laboratory of cryptosporidiosis. Authors concluded that cryptosporidiosis occurred predominantly in young children (<5 years old) and in young adults, mainly during late summer. Most patients were immunocompetent (60%) and deaths were only reported in immunocompromised patients. In addition, *C. parvum* was largely predominant in the French population, with an over representation of *C. parvum gp60* subtypes IIa and IId, suggesting frequent zoonotic transmission.

To evaluate the genetic diversity of *Cryptosporidium* isolates, Zhang et al. [8] analyzed the sequence characteristics of the cgd6_5520-5510 gene, which is divergent in different *C. parvum* subtypes and which encode insulinase-like proteases. The authors found the existence of multiple copies of the gene in some *C. parvum* subtype families supporting the potential involvement of the insulinase-like protease in the broad host range of *C. parvum* IIa and IId subtypes. Interestingly, these sequence types were discordant to sequence types at the *gp60* locus.

Research on the interaction between the intestinal microbiome and *Cryptosporidium* was the focus of the article published by Charania et al. [9]. Following antibiotic treatment of *Cryptosporidium*-infected mice, a significant increase in severity of cryptosporidial infection and in gut permeability was emphasized simultaneously with the shift in bacterial infection. In addition, specific alterations in the host microbiome were shown to be associated with favorable growth of cryptosporidial parasites. 

The investigation of host-parasite interactions is crucial for a better understanding of the mechanisms of parasitic infection. In this sense, Xu et al. [10] provided evidence of the role of INS-15, an insulinase-like protease in the process of *C. parvum* invasion. The INS-15 protein was proved to be expressed in the mid-anterior region of sporozoites and the area of merozoites opposite to the nucleus. Moreover, anti-INS-15 domain I antibodies reduced the invasion of *C. parvum* sporozoites by over 40%. For their part, Cui et al. [11] investigated gene expression patterns, protein localization in developmental stages, and in vitro neutralization characteristics of Cpgp40/15 and Cpgp40, two zoite surface proteins proposed to be involved in attachment and invasion of host cells. Indirect immunofluorescence assay indicated that Cpgp40/15 was associated with the parasitophorous vacuole membrane during *Cryptosporidium* intracellular development. Both anti-Cpgp40/15 and anti-Cpgp40 antibodies were able to neutralize *C. parvum* infection in vitro. Guesdon et al. [12] in their study investigated the role of Cathelicidin Related Antimicrobial Peptide (CRAMP), an antimicrobial peptide part of the innate immune response, which plays a major role in the control of the acute phase of the infection. Briefly, sporozoites were shown to be sensitive to CRAMP antimicrobial activity. However, during *C. parvum* infection, the intestinal expression of CRAMP was significantly decreased, even if the depletion did not aggravate the infection. On the other hand, the exogenous administration of CRAMP reduced the parasite burden of animals. These observations suggested that *C. parvum* impaired the production of CRAMP, subverting the host response. The review by Sawant et al. [13] described the potential causal link between *Cryptosporidium* infection and digestive cancer, with particular emphasis on colon cancer based on available experimental and clinical data. The current knowledge about the potential mechanisms contributing to cell transformation was also highlighted.

The range of tools to investigate *Cryptosporidium* infection has considerably increased in the last few years as detailed by Gunasekera et al. [14]. These authors reviewed the recent advances in *Cryptosporidium* culturing techniques using three-dimensional intestinal models and both mouse and human-derived intestinal organoids and stated that this new culturing technology might be a promising solution to a major barrier in *Cryptosporidium* research.

Altogether, these papers represent a significant contribution to a better understanding of the epidemiology, circulation and virulence of *Cryptosporidium* and stimulate new fields of research about this parasite. We hope that the readers enjoy this Special Issue.
